# Antibacterial Activity of Root Repair Cements in Contact with Dentin—An Ex Vivo Study

**DOI:** 10.3390/jfb14100511

**Published:** 2023-10-11

**Authors:** Andreas Koutroulis, Håkon Valen, Dag Ørstavik, Vasileios Kapralos, Josette Camilleri, Pia Titterud Sunde

**Affiliations:** 1 Section of Endodontics, Institute of Clinical Dentistry, Faculty of Dentistry, University of Oslo, 0317 Oslo, Norway; d.s.orstavik@odont.uio.no (D.Ø.); vasileios.kapralos@odont.uio.no (V.K.); 2 Nordic Institute of Dental Materials (NIOM), 0855 Oslo, Norway; hakon.valen@niom.no; 3 School of Dentistry, Institute of Clinical Sciences, College of Medical and Dental Sciences, University of Birmingham, Birmingham B15 2TT, UK; j.camilleri@bham.ac.uk

**Keywords:** bacterial viability, characterisation, endodontic cement, perforation repair, root-end filling material, tricalcium silicate

## Abstract

This study assessed the antibacterial characteristics of the dentin/material interface and dentin surfaces exposed to experimental hydraulic calcium silicate cement (HCSC) with or without bioactive glass (BG) replacement (20% or 40%) or mixed with a silver nanoparticle (SNP) solution (1 or 2 mg/mL), and Biodentine, TotalFill BC RRM putty and Intermediate Restorative Material (IRM). Human root dentin segments with test materials were assessed at 1 or 28 days. In one series, the specimens were split to expose the dentin and material surfaces. A 24 h direct contact test was conducted against three-day established *Enterococcus faecalis* and *Pseudomonas aeruginosa* monospecies biofilms. In another series, the dentin/material interface of intact specimens was exposed to biofilm membranes for 3 days and the antibacterial activity was assessed via confocal microscopy. The interface was additionally characterised. All one-day material and dentin surfaces were antibacterial. Dentin surfaces exposed to HCSC with 40% BG-replacement, Biodentine and IRM had decreased antibacterial properties compared to those of the other cements. The HCSC mixed with a 2 mg/mL SNP solution had the highest antimicrobial effect in the confocal assay. The interfacial characteristics of HCSCs were similar. The test materials conferred antibacterial activity onto the adjacent dentin. The BG reduced the antibacterial effect of dentin exposed to HCSC; a 2 mg/mL SNP solution increased the antibacterial potential for longer interaction periods (three-day exposure).

## 1. Introduction

Hydraulic calcium silicate-based cements (HCSCs) are a material category with applications in many clinical endodontic procedures [1,2,3]. The hydration reaction of calcium silicate, forming calcium silicate hydrate gel and calcium hydroxide, is the principal chemical reaction in these materials [4]. Their main beneficial properties are the hydraulic nature and the release of calcium hydroxide; the former may enable them to withstand exposure to the wet clinical environment, while the latter’s potential role in providing an alkaline-mediated antimicrobial effect has been described [5]. Ionic leaching has also been linked to the materials’ osteogenic potential in contact with bone tissue [6].

Several formulations have been launched since the introduction of the first commercialised HCSC in the 1990s, Mineral Trioxide Aggregate (MTA) [7]. Biodentine (Septodont, Saint Maur-des-Fosses, France) and TotalFill formulations (FKG Dentaire Sar, La Chaux-de-Fonds, Switzerland) are representative materials of the newer generations of HCSCs. These materials consist mainly of pure calcium silicates, inert fillers as radio-opacifiers, and other additives. Biodentine contains a plasticiser for the improvement of the handling properties, and calcium chloride and calcium carbonate to accelerate the setting time and control the hydration reaction, respectively [8]. TotalFill products are applied without previous mixing with a liquid component, needing the moisture available in the application field for hydration instead. They also contain calcium sulphate as a moderator of the hydration reaction, as well as calcium phosphate cement [9].

The interactions of HCSCs with the clinical environment vary depending on the specific procedure for which the materials are applied [4]. When used for retrograde filling or perforation repair, the materials should establish a barrier between the periradicular tissues and the root canal system to prevent infections [10]. Bacteria that have survived insufficient root canal disinfection procedures may create pathways towards the extraradicular area [11]. The role of dentin in this process is also important since bacteria can colonise dentinal tubules [12], especially when a root resection is performed and the tubules are exposed to the periradicular area [1]. The interactions between the material and dentin at the interfacial area are therefore crucial for the establishment of a physically adequate seal, and the release of antibacterial constituents that may aid in reducing the risk of reinfection.

The incorporation of compounds that could enhance the adaptation of HCSC to dentin would be beneficial in securing a physical barrier. Bioactive glass (BG) formulations are calcium sodium phosphosilicates with a re-mineralisation capacity; in contact with the phosphate-containing host tissue, the BG reacts by releasing calcium and sodium ions, with the subsequent formation of a silica-rich layer, which serves as a substrate for hydroxyapatite formation [13]. Their apatite-forming ability has been exploited upon incorporation into resin composites [14], endodontic sealers [15], HCSCs, and, particularly, Biodentine [16,17]. At the same time, modifications towards the enhancement of target cement properties may negatively affect other basic material characteristics [18].

From a different angle, enhancing the bactericidal potential of HCSCs through the addition of antibacterial constituents could be beneficial given that reports on their current efficacy seem somewhat inconclusive [4]. Candidates for such additions include silver nanoparticles (SNPs), which can impair the bacterial cell membrane, disrupt protein synthesis, inhibit DNA replication and eventually cause cell death [19,20]. In fact, their use has been explored in several aspects of root canal disinfection as irrigation solutions, medicaments, and for incorporation in endodontic cements [21].

The aim of the current study was to assess the antibacterial characteristics of the dentin/material interface of prototype HCSCs with or without addition of silver nanoparticles or bioactive glass in comparison with the commercial materials Biodentine, TotalFill BC RRM Putty and Intermediate Restorative Material. The null hypotheses tested were that the antibacterial properties of cements upon the dentin/material interface will not differ for modified HCSCs and that contact with cements will not contribute to any dentin antibacterial effect.

## 2. Materials and Methods

### 2.1. Test Materials

The following chemicals were used to make up the prototype cements: tricalcium silicate cement (TCS; American Elements, Los Angeles, CA, USA); zirconium oxide (ZO; Koch-Light Laboratories, Colnbrook, Bucks, UK); ultrapure water (water; Elix Essential 5 UV Water Purification System, Merck KGaA, Darmstadt, Germany); bioactive glass 45S5 (BG; Cas No: 65997–17-3, 10 μm particle size, Mo-Sci Corporation, Rolla, MO, USA); and silver nanopowder (100 nm particle size, Sigma-Aldrich, Gillingham, UK) dispersed in water at a concentration of 1 mg/mL or 2 mg/mL SNP-concentration [22].

The tested materials were as follows:

- ΤΖ-base: 80% *w*/*w* TCS and 20% *w*/*w* ZO mixed with water;
- TZ-bg20: ΤΖ-base with 20% *w*/*w* BG replacement of TCS mixed with water;
- TZ-Bg40: ΤΖ-base with 40% *w*/*w* BG replacement of TCS mixed with water;
- TZ-Ag1: TZ-base mixed with 1 mg/mL SNP solution (0.35 mg SNP addition per 1 g of TZ-base powder);
- TZ-Ag2: TZ-base mixed with 2 mg/mL SNP solution (0.7 mg SNP addition per 1 g of TZ-base powder);
- Biodentine (Septodont, Saint Maur-des-Fosses, France);
- TotalFill Root Repair Material—BC RRM Putty (TotalFill; FKG Dentaire, La Chaux-de-Fonds, Switzerland);
- Intermediate Restorative Material (IRM; Dentsply Sirona, Charlotte, NC, USA).

Before placement, the prototype materials were hand-spatulated at a 0.35 liquid/powder ratio and the commercial materials were handled according to the manufacturers’ instructions.

### 2.2. Preparation of Root Segments

**Figure 1:**
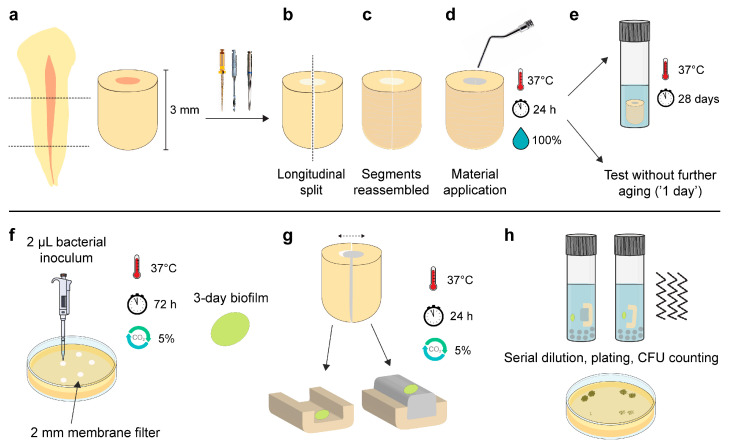
Schematic representation of the antibacterial assay described by Kapralos et al. [23]. (**a**) Root segments (3 mm length) were obtained and the canal spaces were enlarged with a series of rotary instruments and post drills. Subsequently, (**b**) the root segments were longitudinally split with a cut-off instrument and (**c**) were reassembled and held tightly with plastic membrane. (**d**) The materials were applied inside the clamped segments and were allowed to set for 24 h. (**e**) They were then either used directly for antibacterial testing or immersed in Hank’s balanced salt solution for 28 days prior to testing. (**f**) For antibacterial testing, three-day monospecies biofilms of *Enterococcus faecalis* and *Pseudomonas aeruginosa* were formed upon membrane filters. (**g**) The two parts of the root segments were carefully separated and the biofilm membranes were placed in direct contact with the material surface and the dentin surface. (**h**) Following a 24 h contact period, each part of the segment with the respective biofilm membrane was immersed in tubes containing phosphate-buffered saline and glass beads, vigorously vortexed and plated after serial dilutions. Colony-forming units (CFUs) were counted the next day. Figure 1e,f,h was partly generated using Servier Medical Art (Servier, Suresnes, France), licensed under a Creative Commons Attribution 3.0 unported license.

**Figure 2:**
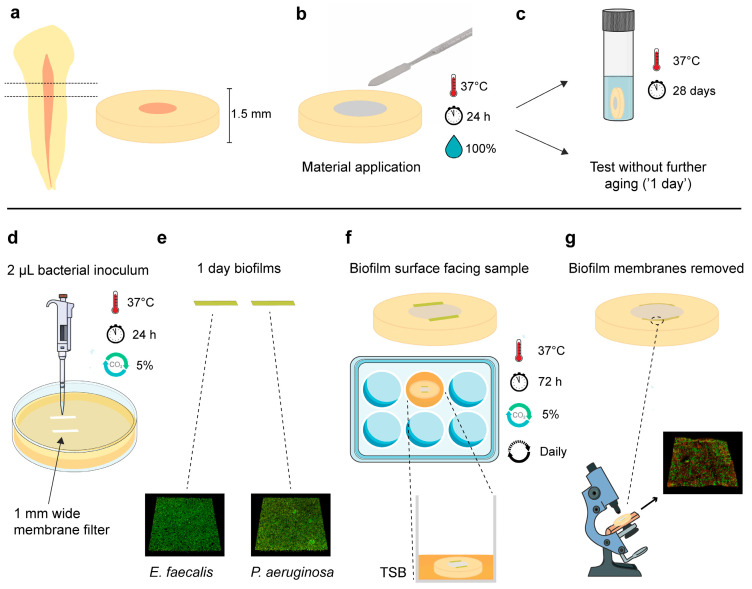
Schematic representation of the 3-day biofilm assay upon the dentin/material interface. (**a**) Root segments (1.5 mm thick) were acquired. (**b**) Upon preparation of the root segments, the materials were compacted in the root canal space. (**c**) The specimens were allowed to set for 24 h, and, consequently, they were either immediately tested (1 day), or immersed in Hank’s balanced salt solution for a total period of 28 days prior to testing. (**d**) Membrane filters positioned upon agar plates were inoculated with *Enterococcus faecalis* or *Pseudomonas aeruginosa*. (**e**) One-day monospecies biofilms were obtained. (**f**) Biofilm membranes were positioned along the dentin/material interface, with the biofilm surface in direct contact with the sample. The samples were incubated with tryptic soya broth (TSB) for 3 days and transferred to new wells with a fresh medium daily. (**g**) The biofilm membranes were removed and the dentin/material interface area below was stained with a bacterial viability kit and imaged with confocal laser scanning microscopy. Figure 2c,d,f,g was partly generated using Servier Medical Art (Servier, Suresnes, France), licensed under a Creative Commons Attribution 3.0 unported license.

The Regional Committee for Medical and Health Research Ethics evaluated the project and deemed that no approval was required to conduct the study (REC Ref. 283811). Extracted human teeth were collected from a tooth biobank (REC Ref. 2013/413). The teeth’s coronal parts and apical 2 mm were sectioned with a precision cutting machine (IsoMet Low Speed; Buehler, Lake Bluff, IL, USA). Two forms of root segments preparations were acquired:

1. 3 mm length (Figure 1a);
2. 1.5 mm thickness (Figure 2a).

The 3 mm long root segments were instrumented with ProTaper rotary files (Dentsply Maillefer, Tulsa, OK, USA) and fibre post drills (3M, St. Paul, MN, USA) up to size 5. A saline solution was used for irrigation. Subsequently, they were carefully longitudinally split using the cutting machine (Figure 1b).

The 1.5 mm thick root segments were carefully enlarged with the fibre post drills to ensure that the samples had similar internal diameters in the root canal area.

The internal surfaces of all root segments were treated with 17% ethylenediaminetetraacetic acid (Pulpdent, Watertown, MA, USA) for 5 min and subsequently with saline solution. The specimens that were used for antibacterial assays were autoclaved in vials with water at 121 °C for 21 min. Finally, the split root segments were reassembled and clamped with Parafilm M (Bemis; Thermo Fisher Scientific, Waltham, MA, USA) wrapped around them (Figure 1c).

### 2.3. Assessment of Antibacterial Effect

Sterile equipment was used for all the procedures.

#### 2.3.1. Material Application and Testing Periods

The materials were compacted in root segments (Figure 1d and Figure 2b) and were allowed to set for 24 h at 37 °C, wrapped with a wet gauze. The next day, they were either tested (‘1-day samples’) or immersed in 1 mL of Hank’s balanced salt solution (HBSS; H6648, Sigma-Aldrich, Gillingham, UK) and stored for a total of 28 days at 37 °C before they were retrieved and tested (‘28-day samples’) (Figure 1e and Figure 2c). Twelve specimens per subgroup (n = 12) were used in each experiment (f = 0.25; α = 0.05; power = 95%), as indicated by an a priori power analysis estimation using G*power 3.1.9.2 (Heinrich Heine University, Düsseldorf, Germany).

#### 2.3.2. Test Bacteria

*Enterococcus faecalis* OG1RF American Type Cell Culture Collection (ATCC; Manassas, VA, US) 47077 and *Pseudomonas aeruginosa* ATCC 9027 were grown overnight in tryptic soya broth (TSB; Sigma-Aldrich, Gillingham, UK) at 37 °C in a 5% CO_2_-supplemented atmosphere. A bacterial inoculum (≈10^8^ colony forming units (CFUs)/mL) for each species was prepared the next day following centrifugation of the overnight culture and resuspension in TSB.

#### 2.3.3. Antibacterial Effect against Three-Day Established Biofilms

A previously described protocol was employed to investigate the direct antimicrobial efficacy of materials after contact with dentin and any potential residual antibacterial activity in the dentin surfaces [23]. Three-day monospecies *E. faecalis* and *P. aeruginosa* biofilms were cultured on circular membrane filters cut to a 2 mm diameter (MF-Millipore; 0.45 μm pore size, Merck KGaA, Darmstadt, Germany). The membranes were positioned upon TSB agar plates and received a 2 μL bacterial inoculum. After 72 h of incubation at 37 °C and in a 5% CO_2_-supplemented atmosphere, the membranes were retrieved (Figure 1f).

The root segments were unwrapped from the parafilm and carefully separated, resulting in a dentin/material segment, referred to as ‘material surface’, and a material-free dentin segment, referred to as ‘dentin surface’. Scanning electron microscopy (TM4000Plus II, Hitachi, Tokyo, Japan) with accompanying energy dispersive X-ray analysis was performed at this point in two additional root segments from each material to confirm that the dentin surfaces were not covered with material remnants (Figure S1).

The biofilm membranes were carefully positioned in direct contact with the material and dentin surfaces (Figure 1g). They were additionally positioned upon dentin surfaces that had not come into contact with any material, serving as negative control (n = 12/bacterium). The samples were incubated for 24 h at 37 °C in a 5% CO_2_-supplemented atmosphere. Subsequently, they were transferred to tubes containing glass beads and 2 mL of phosphate-buffered saline and vortexed. After 10-fold serial dilutions, the samples from the liquid were plated on TSB agar plates, and the bacterial counts were assessed the following day (Figure 1h).

#### 2.3.4. Biofilm Assay at the Dentin/Material Interface

Following the specified ageing periods of 1 or 28 days, the intact root segments (1.5 mm thick) were carefully ground with silicon carbide paper discs (Grit 500; Struers, Rotherham, UK) to ensure a flat surface, and subsequently rinsed with sterile water. They were then dried with filter paper and sterilised for 20 min on each side under UV light.

One-day monospecies *E. faecalis* and *P. aeruginosa* biofilms were cultured on rectangular membrane filters (1 mm wide) with the same method described for the three-day circular biofilm membranes above (Figure 2d,e). Consequently, the rectangular biofilm membranes were carefully positioned along the length of the dentin/material interface of each sample (two membranes per root disc), with the biofilm surface facing towards the sample. The specimens were transferred into 24-well plates and inoculated with 800 μL TSB (Figure 2f). They were incubated at 37 °C in a 5% CO_2_-supplemented atmosphere for 72 h. Every 24 h, the samples were transferred into a new well with fresh TSB.

After this period, the samples were retrieved and gently immersed in sterile pure water. The biofilm membranes were carefully removed, and the samples were then stained with a 50 μL dye solution of LIVE/DEAD BacLight Bacterial Viability Kit (Invitrogen, Eugene, OR, USA) for 20 min in a dark room. Subsequently, they were immersed in water to remove the excess of stain and imaged using a confocal laser scanning microscope (CLSM; Olympus FluoView FV1200, Olympus, Tokyo, Japan). Three microscopic confocal volumes from random areas upon the dentin/material interface where the biofilm membranes were previously positioned were acquired from each sample using a 60× water immersion lens, 1 μm step-size and a format of 480 × 480 pixels (Figure 2g). For a better orientation in the area of interest and threshold application in the CLSM, samples that were not subjected to a biofilm challenge were additionally imaged (Figure S2). Polyester coverslip discs (13 mm, Thermanox Coverslips, Thermo Fisher Scientific, Waltham, MA, USA) were used as additional negative control substrates to confirm biofilm formation upon the areas where the rectangular biofilm membranes were applied. The percentage of injured/dead cells and the total biofilm volume (μm^3^) were assessed with Bioimage_L software (v.3.0, Department of Oral Biology, Malmo University, Sweden) [24].

### 2.4. Microstructural and Chemical Analysis of the Interface

The materials (n = 3/group) were compacted in the root segments (1.5 mm thick) as specified in the section above (Figure 2b) and were stored in HBSS at 37 °C for 28 days (Figure 2c). Subsequently, the samples were retrieved, dried in filter paper, vacuum-desiccated and embedded in auto-polymerising epoxy resin (Epoxyfix; Struers, Rotherham, UK). They were then ground in water and polished with a series of silicon carbide papers and a polishing cloth (Struers, Rotherham, UK). A diamond suspension (DP-1 μm; Struers, Rotherham, UK) was used for the polishing procedure. The material to dentin interface was imaged with a scanning electron microscope (SEM; TM4000Plus II, Hitachi, Tokyo, Japan) under backscatter mode following the sputter-coating of specimens with gold (Agar Scientific, Essex, UK). Energy dispersive X-ray (EDX) analysis was performed and elemental maps across the interfaces were created.

### 2.5. Data Analysis

The data were assessed for normality with the Shapiro–Wilk test as well as histogram and q-q plot evaluation. Due to the absence of normal distribution, the data from confocal microscopy were subjected to square root transformation, while the data from the CFU-based direct contact experiments were log-transformed and expressed as log(CFU + 1)/mL.

For data from the CFU-based experiment of dentin surfaces and those from confocal microscopy, univariate general linear models were fitted for the following factors: ‘Materials’, ‘Ageing period’, and ‘Bacterium’. The normality of residuals following each analysis was assessed with q-q plots. Pairwise comparisons were conducted with the Bonferroni correction. Additionally, a Student’s t-test was conducted for the comparison of dentin surfaces with the negative control.

For data from the CFU-based experiment of material surfaces, an analysis was conducted with non-parametric tests as, despite the log-transformation of the data and due to the presence of many zero values within groups, none of the applied generalised linear models could provide an adequate fit. We therefore decided to perform analyses for materials within the same ageing period and for the same test bacterium with the Kruskal–Wallis test and post hoc methods adjusted using the Bonferroni correction. Mann–Whitney U tests were used in the case of comparing the effect of the ageing period within the same material and test bacterium, as well as for comparisons with the negative control.

All analyses were conducted with SPSS software 29.0 (IBM, Armonk, NY, USA). The significance level was set as α = 0.05.

## 3. Results

### 3.1. Antibacterial Investigation

#### 3.1.1. Independent Assessment of Material and Dentin Surfaces

**Figure 3:**
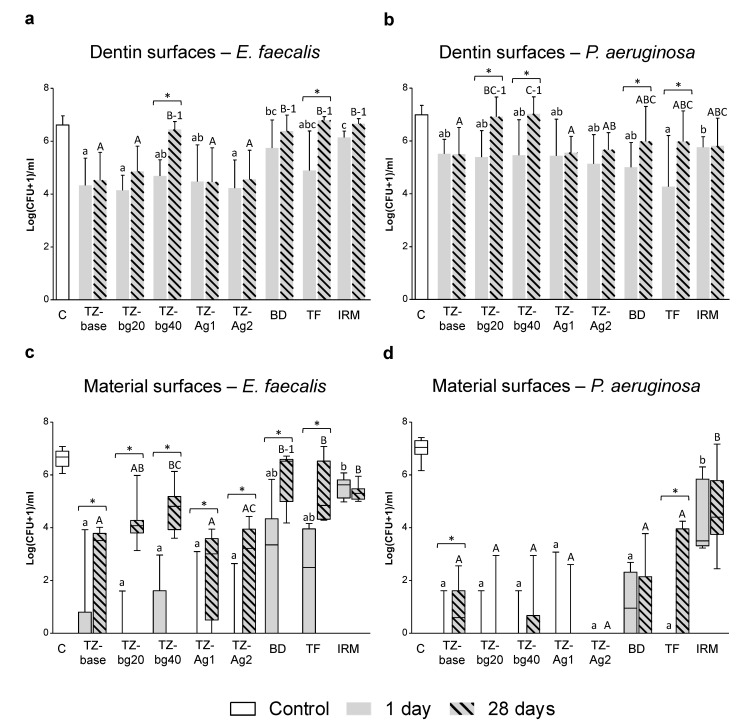
Antibacterial activity of dentin and material surfaces from split root segments. (**a**,**b**) Mean log(CFU + 1)/mL and standard deviation of *Enterococcus faecalis* or *Pseudomonas aeruginosa*, respectively, following direct exposure of 3-day monospecies biofilms with dentin surfaces. (**c**,**d**) Box and whiskers plots (minimum value, lower quartile, median value, upper quartile and maximum value) for bacterial survival following exposure to material surfaces from the root segments. Different lowercase letters per graph indicate statistically significant differences among the 1-day samples. Different capital letters indicate statistically significant differences among the 28-day samples. Brackets with asterisks above bars or boxes indicate a significant difference between 1- and 28-day samples within the same material. Groups with the number ‘1’ did not show any statistical reduction in bacterial counts compared to the control (*p* < 0.05). Abbreviations: C, negative control; BD, Biodentine; TF, TotalFill.

Figure 3 presents the results from the antibacterial testing of dentin and material surfaces.

##### Dentin Surfaces

Upon accounting for the effect of the ‘Ageing period’ and ‘Bacterium’, the overall antibacterial activity of the dentin surfaces following contact with TZ-bg40, Biodentine and IRM was significantly lower than that of the respective specimens from TZ-base, TZ-Ag1 and TZ-Ag2 (*p* < 0.001 for comparisons with TZ-bg40 and IRM; *p* < 0.05 for comparisons with Biodentine).

More specifically, the dentin surfaces that had been in contact with the prototype HCSC for 1 or 28 days presented significantly reduced *E. faecalis* colonies compared to the control (*p* < 0.05), except for those in contact with TZ-bg40 for 28 days. The dentin surfaces exposed to Biodentine, TotalFill and IRM presented reduced *E. faecalis* colonies only after 1 day of application (*p* < 0.05) (Figure 3a).

All samples caused a reduction in bacterial counts against *P. aeruginosa* compared to the control (*p* < 0.05), except for those in 28-day contact with TZ-bg20 or TZ-bg40 (Figure 3b).

##### Material Surfaces

All prototype HCSCs significantly reduced the colonies of both bacterial species after 1 and 28 days of material ageing (*p* < 0.05). TZ-base, TZ-Ag1 and TZ-Ag2 caused a higher logCFU-reduction in *E. faecalis* biofilms than that of the commercial cements in the 28-day test period (*p* < 0.01). The bactericidal effect of both the prototype and the commercial HCSCs against *E. faecalis* was reduced between the 1 and 28 days samples (*p* < 0.05). A decline in antibacterial effectiveness against *P. aeruginosa* over time was observed for TZ-base and TotalFill (Figure 3c,d).

#### 3.1.2. Biofilm Assay upon the Dentin/Material Interface

**Figure 4:**
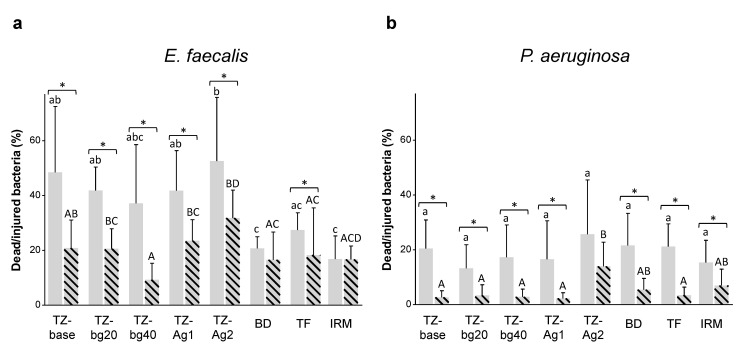
Mean and standard deviation of dead/injured (**a**) *Enterococcus faecalis* or (**b**) *Pseudomonas aeruginosa* cells (%) prior to root square transformation of the data. Different lowercase letters indicate statistically significant differences among the 1-day samples. Different capital letters indicate statistically significant differences among the 28-day samples. Brackets with asterisks above bars in the graphs indicate a significant difference between the 1- and 28-day samples within the same material (*p* < 0.05).

**Figure 5:**
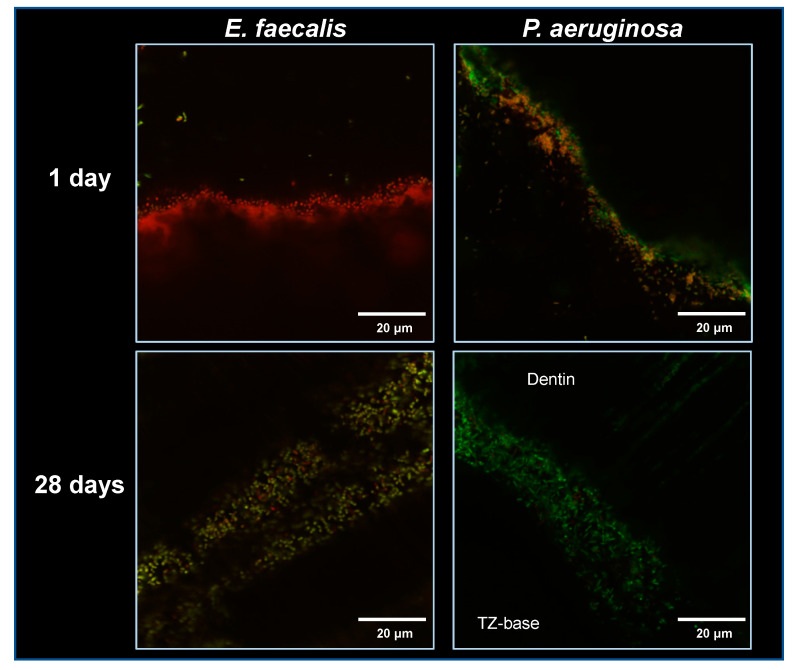
Representative confocal laser scanning microscope images from z stack series of biofilms upon the dentin/material interface for TZ-base following staining with bacterial viability kit. Dead-injured bacteria were stained red, and live ones appear as green. All images had the same magnification (60×) and pixel format (512 × 512) and each image represents an area of 88 × 88 μm.

Figure 4 shows the results of the percentage of dead/injured bacteria, and Table 1 presents the total biofilm volume for *E. faecalis* and *P. aeruginosa*. Figure 5 shows representative confocal laser scanning microscope images from the test samples.

A significantly lower proportion of dead/injured bacteria was overall observed in the 28-day samples compared to the 1-day specimens (*p* < 0.001). TZ-Ag2 had an overall significantly higher antibacterial effect than the other tested materials (*p* < 0.05; Figure 4). The *P. aeruginosa* specimens were less susceptible to the antibacterial effect of materials than *E. faecalis* (*p* < 0.001).

The *E. faecalis* biofilms upon the dentin/material interface were significantly thicker compared to *P. aeruginosa* (*p* < 0.001). Taking into account the factors ‘Ageing period’ and ‘Bacterium’, the biofilm volume was not affected by the material type (*p* > 0.05). It was, nevertheless, overall larger in the 28-day test root segments than in the 1-day ones (*p* < 0.001).

### 3.2. Characterisation of the Dentin/Material Interface

**Figure 6:**
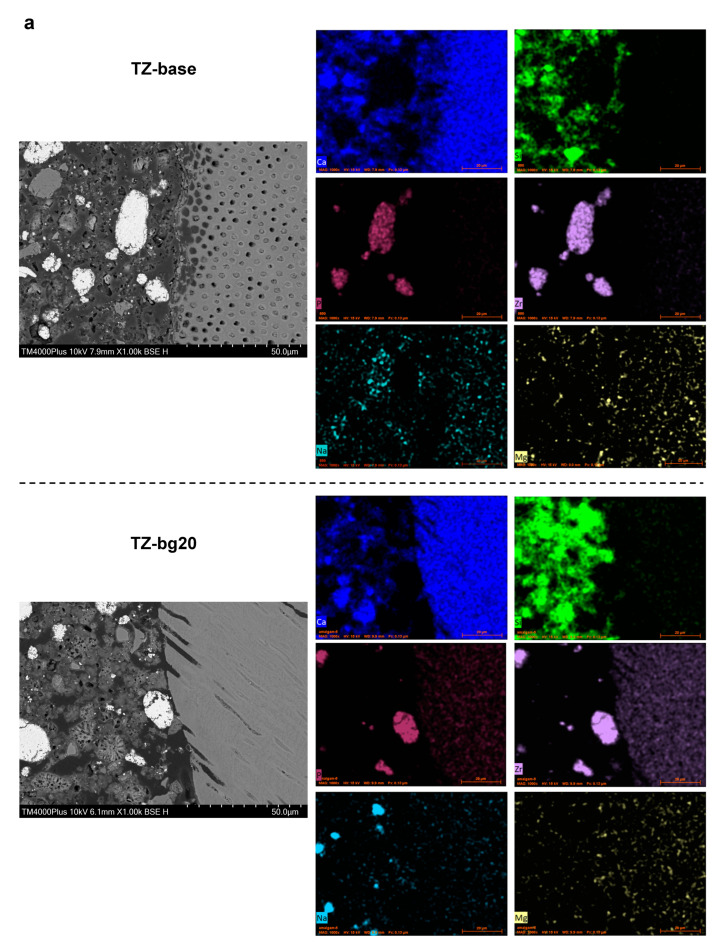

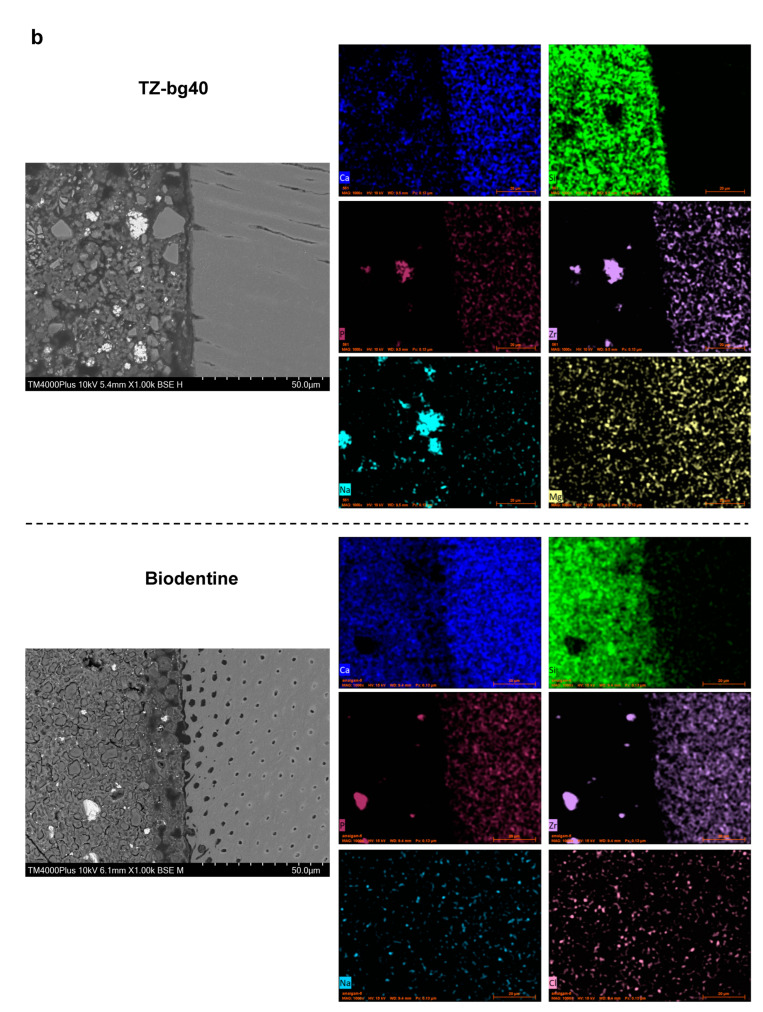

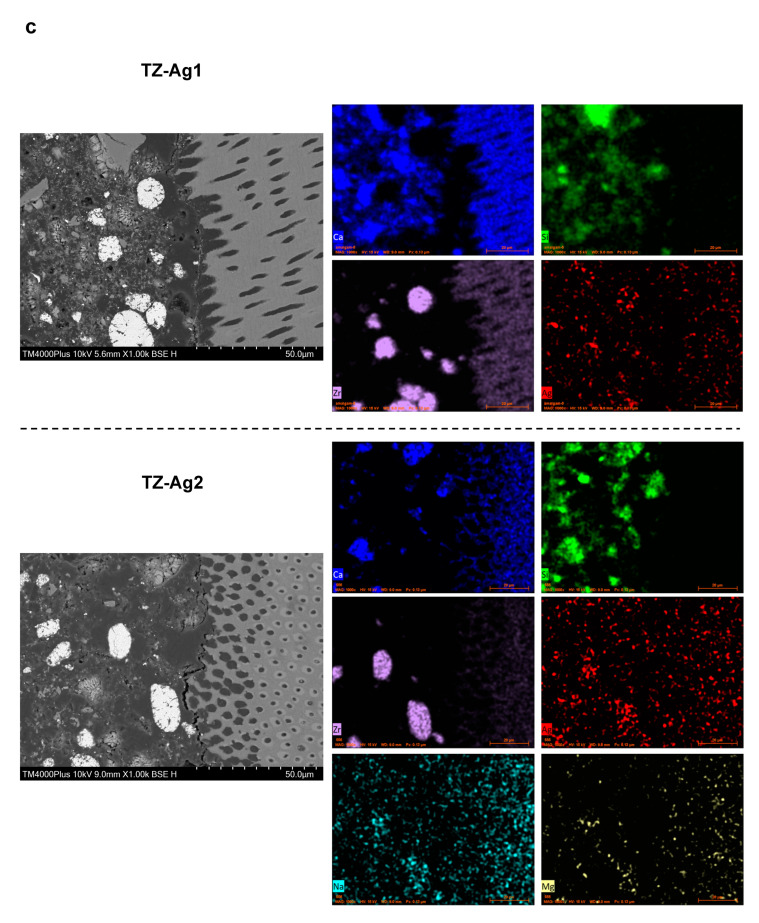

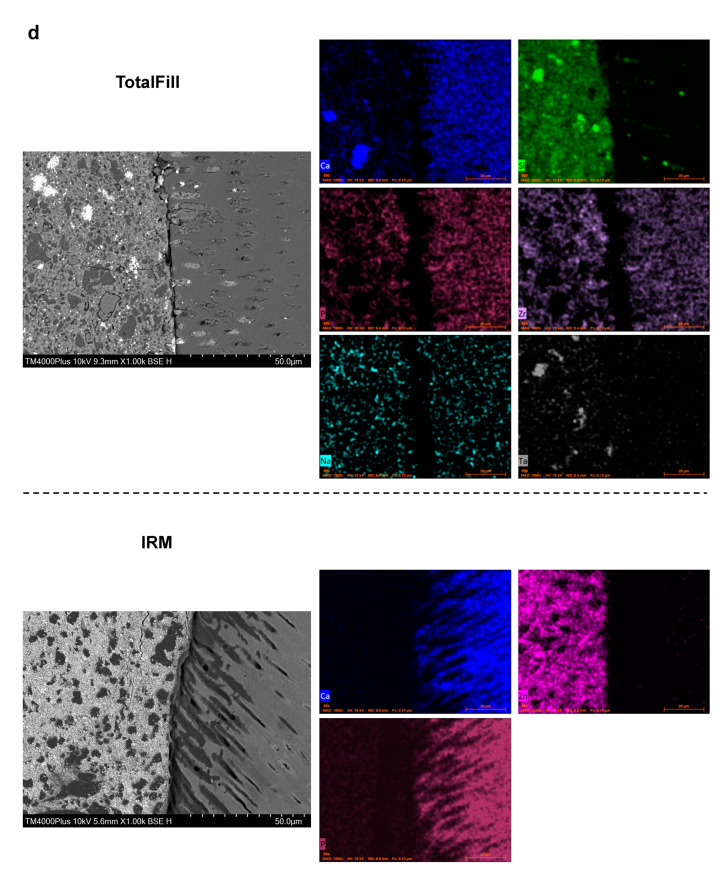
Backscatter scanning electron micrographs of the dentin/material interface (1000×) accompanied by elemental maps mainly of calcium (blue), silicon (green), and zirconium (purple), as well as sodium (light blue), phosphate (plum), magnesium (yellow), chlorine (lilla), silver (red), tantalum (grey) or zinc (Tyrian purple) for (**a**) TZ-base and TZ-bg20, (**b**) TZ-bg40 and Biodentine, (**c**) TZ-Ag1 and TZ-Ag2, (**d**) TotalFill and IRM. An overlap in the peaks of phosphate and zirconium exists.

Indicative microscopic images with accompanying elemental maps of the dentin/material interfacial zones are presented in Figure 6. Silicon was the most prevalent element in the interface of calcium silicate cements, especially for TZ-bg40, followed by Biodentine and TotalFill. Calcium was also evident in all HCSCs. The migration of silver into dentin was evident in the SNP-containing cements, especially in TZ-Ag2. Gaps of varying width between the material and dentin surface occurred in all samples.

## 4. Discussion

The current study assessed the antibacterial properties of prototype and commercial HCSCs at the dentin/material interface. All tested cements conferred an antibacterial activity to the adjacent dentin tested 1 day following material application. Changes in the antibacterial efficiency were observed with material modifications across the different assays such as a reduction upon replacement of TCS with 40% BG, or enhancement after the incorporation of 2 mg/mL SNP into the liquid component. The null hypotheses that the antibacterial characteristics of cements would be similar and that dentin exposed to materials would not present any antibacterial potential were therefore rejected.

Reinfection by persisting or newly established microorganisms in the root canal system may occur following retrograde treatment or perforation repair [11]. This makes the antibacterial properties of root repair materials worth investigating [25]. Common commercial HCSCs exhibit varying antibacterial efficacy [4], which is mainly ascribed to the creation of an alkaline microenvironment. Yet, alkalinity may be buffered by tissue fluids, particularly in an acidic inflamed environment [26]. Furthermore, the role of dentin appears to be intricate. From one perspective, dentin also has a buffering capacity [26,27], which can be detrimental to the antibacterial effect [28]. At the same time, due to its complex anatomy, it can act as a reservoir of the ions released by the adjacent cements and mediate an antibacterial effect to the microenvironment through this ionic flow [29].

Considering these aspects, we used SNPs because they may diffuse into dentinal tubules and be subsequently released to enhance the antibacterial activity [30]. Bioactive glass was employed to assess whether it could alter the antibacterial characteristics of HCSC at the interface. The original BG formulation—BG 45S5—was used in the current study [13].

By evaluating the properties of commercial and prototype HCSCs in the same study, a more comprehensive understanding of the effects of incorporating chemical compounds into them can be achieved. HCSCs were assessed in the context of their use for root-end filling and perforation repair procedures, where the materials should provide an adequate seal between the root canal system and the periradicular area [10]. Biodentine and TotalFill are indicated for such applications [31,32]. Finally, the properties of the zinc oxide eugenol-based IRM were also examined as a comparative reference. IRM is an extensively studied cement that is widely employed in retrograde procedures, presenting a success rate similar to that of MTA [33,34].

Two studies have previously used a dentin substrate to evaluate the antibacterial properties of Biodentine [35,36], while, to the best of our knowledge, the antibacterial properties of the putty version of TotalFill have not been tested on a dentin model before. The lack of standardised test protocols for antibacterial testing [37] and limited data from ex vivo models for root repair materials [4] suggest a knowledge gap regarding the essential properties of the most widely used HCSCs. Two distinct dentin models were therefore utilised here to investigate the antibacterial properties at the dentin/material interface.

The materials were tested either 1 or 28 days after placement. It is anticipated that the elution of antimicrobial compounds by HCSCs, specifically hydroxyl ions, would decrease in the long term [38].

The split tooth model, originally designed to assess the residual antimicrobial effect of irrigation solutions and sealers [23], was used here to independently evaluate the antibacterial potential of both the material and the dentin surface at the interface following the designated application periods. Even though dentin does not serve as a substrate for biofilm cultivation in the model, it enables the investigation of the indirect antibacterial potential from the test materials. Our results did in fact demonstrate the transfer of an antimicrobial effect to dentin. However, replacing BG in HCSC resulted in a reduction in this effect after 28 days, especially upon 40% replacement of TCS. Prior studies have demonstrated that the antibacterial properties of a different form of BG were enhanced upon contact with powdered dentin or bone; mineralised tissues appear to facilitate the dissolution of BG, resulting in an increased release of silica and elevated pH levels [39,40,41]. Even though the bactericidal potential of BG is mainly alkaline-related, the current study showed that its effect appeared to be insufficient to compensate for the antibacterial activity lost due to the replacement of tricalcium silicate. This inferior antibacterial potential is also evident in studies where BG undergoes ageing under physiological conditions, as the buffering capacity of the host’s fluids can moderate the alkalisation effect [42,43]. Thus, the beneficial characteristics of BG could be further explored as additions in the cementitious phase of HCSC instead of replacing a portion of the TCS. The potential of BG to enhance the remineralisation process on dentin [44] could be further explored in conjunction with its antibacterial characteristics. Even though the evaluation of the former was not in the scope of the current study, no consistent pattern of coverage of dentinal tubules was observed as being carried out by the BG-containing HCSCs compared to the unmodified cement. Upon assessing model reproducibility, the SEM/EDX observations of the split segments showed the occasional presence of cement particles on the dentin surface, with some of them also penetrating inside the dentinal tubules. The materials affected the elemental composition of dentin segments as evidenced by the spectroscopical scans, yet these findings were not exclusive to the BG-containing cements.

In order to further assess the antibacterial effect of this area under different conditions, an assay of biofilm growth upon the intact dentin/material interface was conducted. The colonisation of this area could lead to reinfection following treatment [45]. Previous studies have investigated this aspect for endodontic sealers [46,47] but it has not been explored for root repair cements. In the current model, biofilms were initially cultured for one day on membrane filters before being exposed to samples. This approach aided in better localising the specific area of interest. The results showed that the addition of 2 mg/mL SNP to prototype HCSC enhanced its antibacterial performance. The release of SNPs in the neighbouring dentin was confirmed with elemental mapping. However, no enhancement of the antibacterial properties was evident in the split tooth model. The combination of the findings from the two ex vivo models in this study suggests that more extended interaction periods might be required for SNPs to fully exert their antibacterial potential, as has been previously indicated [48]. At the same time, the current results complement the previous research on early bacterial adhesion upon the TZ-Ag2 surface, where no significant changes were observed after 1 h of exposure to an *E. faecalis* inoculum [38]. From another perspective, SNP concentrations higher than the ones used in the current study could be explored in order to achieve potentially more efficient bacterial killing. Here, we selected the 2 mg/mL SNP-concentration based on a protocol for sufficient particle dispersion [22]. Attaining an optimal concentration towards the potential clinical usage of SNPs should also be determined through a combined evaluation of their effect on host tissue cells. The discolouration potential is also a concern [21], particularly given the intended release of SNPs into the subsequent tissues, especially dentin.

The improved antibacterial performance of TZ-Ag2 was mainly evident against *P. aeruginosa*. Gram-negative bacteria are reportedly more susceptible to SNPs due to the thinner layer of peptidoglycan in their cell wall [49]. The Gram-positive *E. faecalis* that was tested here is the most frequently studied bacterium in endodontic literature [50]. Even though its direct causal relationship with post-treatment apical periodontitis has been questioned, it is indeed a commonly isolated bacterium from failed endodontic cases [51,52,53]. The Gram-negative *P. aeruginosa* has also been recovered from persistent endodontic infections [53]. Interestingly, the susceptibility of the two bacterial species differed in the experimental assays, indicating the importance of including variations in the methodological models and the test parameters. The cultivation of monospecies biofilms for antibacterial testing is, however, an undisputed limitation in the current study, as it does not replicate the complexity and resistance of multispecies biofilms that are apparent in clinical practice. On the other hand, the utilisation of simplified biofilm models could ensure better reproducibility in the initial assessment of antibacterial properties [50] and provide insights for subsequent testing.

Overall, in both dentin models studied, no significant differences in the antibacterial potential of the two commercial HCSCs, Biodentine and TotalFill, were observed. However, the indirect antibacterial effect of Biodentine on dentin surfaces appeared smaller than that on the unmodified prototype cement. In fact, the antimicrobial activity of dentin surfaces in contact with either TotalFill or Biodentine was reduced from 1 to 28 days. This observation can be attributed to a faster hydration reaction for both commercial HCSCs compared to the experimental formula (TZ-base), even though the reaction of TotalFill is dependent on the moisture available in the environment [9], which could slow down the process. The putty version, however, is reported to react faster than the paste form [9], possibly due to a difference in the particle size [54]. The faster hydration reaction of Biodentine compared to the radiopacified tricalcium silicate cement is mainly ascribed to the addition of calcium carbonate to the former and has been previously documented [8,55]. Once the most significant part of hydration has taken place, less calcium hydroxide is released. Therefore, the buffering capacity of dentin had a greater impact on the commercial materials, probably due to the earlier completion of diffusion of the hydroxyl ions. The antibacterial characteristics of Biodentine and TotalFill formulations have been found to be similar when tested against cariogenic bacteria with the rationale to use these as pulp capping agents [55,56]. The zinc oxide eugenol-based IRM showed an overall steadier yet inferior antibacterial potential in our experiments. Our findings are in accordance with those of Dragland et al. [57], who reported a stable and modest antibacterial behaviour of IRM over time against *E. faecalis,* as well as a gradual release of eugenol in long evaluation periods.

The dentin/material interface was additionally characterised by means of SEM, EDX and elemental mapping to gain a better understanding of the interactions occurring in that area. Sample dehydration, a step that is required prior to SEM observation, and sample-induced shrinkage caused by the constant vacuum environment during observation can both impair the adaptability of HCSCs to dentin [58]. However, a potential material detachment from the dentin surface does not seem to alter the elemental distribution in the two surfaces [59]. The observations of the interfaces did not reveal notable differences among the HCSCs. The SNPs migrated to dentin, while the BG particles were evident in the bulk of the respective BG-containing cements. Studies on the interfacial characteristics of HCSCs in contact with dentin report somewhat conflicting findings. Atmeh et al. [60] reported the formation of an intermediate mineralised layer at the interface, accompanied by tag-like structures within the dentinal tubules that are triggered by HCSC’s rapid local calcium hydroxide release. This ‘mineral infiltration zone’ has been disputed in subsequent studies, which attribute its presence to an artefact induced by the fluorescent dyes used in confocal microscopy [58]. In contrast, one study reports conventional calcium phosphate precipitation in the interfacial gaps from the reaction of calcium from HCSC with phosphorus in the dentin component [59]. Therefore, the occasional tag-like structures within the dentinal tubules, as also sporadically evident in the current study, are regarded as the cement’s gap-filling property rather than a consistent formation of a mineral layer [59].

## 5. Conclusions

This investigation of the antibacterial properties of HCSCs at the dentin/material interface showed residual antibacterial activity in the dentin surfaces. In the split tooth model, the replacement of TCS with 40% BG reduced the bactericidal activity. A 2 mg/mL SNP liquid component mixed with the prototype HCSC demonstrated an improved antibacterial potential during a three-day biofilm assay at the dentin/material interface, although the effect was relatively small.

## Figures and Tables

**Table 1:** Mean and standard deviation of *Enterococcus faecalis* or *Pseudomonas aeruginosa* biofilm volume (μm^3^) (%) prior root square transformation of the data. Different lowercase letters indicate statistically significant differences among the 1-day samples. Different capital letters indicate statistically significant differences among the 28-day samples. Different numbers indicate a statistically significant difference within the same material and test bacterium between the two ageing periods (*p* < 0.05). Abbreviations: BD, Biodentine; TF, TotalFill.

Total Biofilm Volume (μm^3^)									
		TZ-Base	TZ-bg20	TZ-bg40	TZ- Ag1	TZ- Ag2	BD	TF	IRM
*E. faec.*	**1 day**	59,931 (42,448) ^a−1^	35,408 (16,886) ^a−1^	51,258 (46,555) ^a−1^	43,023 (25,834) ^a−1^	45,676 (47,451) ^a−1^	38,514 (33,513) ^a−1^	71,417 (32,892) ^a−1^	59,927 (65,753) ^a−1^
	**28 days**	78,597 (38,789) ^A,B−1^	64,425 (35,105) ^A,B−1^	125,866 (13,320) ^B−2^	66,004 (45,395) ^A,B−1^	55,959 (42,533) ^A,B−1^	30,916 (9190) ^A−1^	94,330 (67,094) ^B−1^	58,524 (45,241) ^A−1^
*P. aerug.*	**1 day**	21,419 (18,042) ^a−1^	31,569 (21,395) ^a−1^	36,863 (29,436) ^a−1^	44,020 (40,201) ^a−1^	37,925 (40,419) ^a−1^	41,428 (22,880) ^a−1^	47,609 (26,390) ^a−1^	41,750 (30,932) ^a−1^
	**28 days**	52,284 (29,498) ^A−2^	37,383 (41,044) ^A−1^	30,143 (23,320) ^A−1^	40,528 (24,824) ^A−1^	53,708 (22,489) ^A−1^	44,770 (23,916) ^A−1^	44,612 (28,209) ^A−1^	31,252 (15,382) ^A−1^

## Data Availability

The data that support the findings of this study are available from the corresponding author upon reasonable request.

## Supplementary Materials

The following supporting information can be downloaded at: https://www.mdpi.com/article/10.3390/jfb14100511/s1, Figure S1: Indicative scanning electron microscope (SEM) images of dentin surfaces from the split tooth block model accompanied by energy dispersive X-ray analyses (EDX) of selected regions or elemental mapping. The images suggest that no complete coverage of the dentin surface by material remnants takes place following the separation of the two parts of the root segment. (a) Root surfaces treated with 17% ethylenediaminetetraacetic acid (EDTA) and with no application of a material (control specimens) were assessed to detect the main elements as well as the trace ones depicted in dentin segments following EDTA pre-treatment. (b) SEM images and EDX analyses from dentin surfaces previously in contact with TZ-base, TZ-bg20 and TZ-bg40. Some zirconium oxide particles, as well as cement particles, can be sporadically depicted. (c) Dentin surfaces and elemental maps for TZ-Ag1 and TZ-Ag2. Some agglomerated silver nanoparticles could also be depicted (red arrow). (d) SEM images and EDX analyses from dentin surfaces previously in contact with Biodentine, TotalFill and IRM. Material residues are mainly depicted inside the dentin tubules; Figure S2: Representative confocal laser scanning microscope images (60×) for orientation upon the dentin/material surface and optimisation of threshold options. The specimens were not exposed to any bacteria. Biodentine was used for these internal controls.
