# Supplementary material for: Antibacterial Activity of Root Repair Cements in Contact with Dentin—An Ex Vivo Study
Source: J Funct Biomater. 2023 Oct 11;14(10):511. doi: 10.3390/jfb14100511 (PMC10607193; doi:10.3390/jfb14100511)
Supplement: Supplementary file 1 [file jfb-14-00511-s001.zip › jfb-2607297-supplementary.pdf]

Journal of *Functional Biomaterials*

MDPI

Supplementary Materials

Antibacterial Activity of Root Repair Cements in Contact with Dentin—An Ex Vivo Study

Andreas Koutroulis [1,*], Håkon Valen [2], Dag Ørstavik [1], Vasileios Kapralos [1], Josette Camilleri [3] and Pia Titterud Sunde [1,*]

[1] Section of Endodontics, Institute of Clinical Dentistry, Faculty of Dentistry, University of Oslo, P.O. Box 1109, Blindern, 0317 Oslo, Norway; d.s.orstavik@odont.uio.no (D.Ø.); vasileios.kapralos@odont.uio.no (V.K.)

[2] Nordic Institute of Dental Materials (NIOM), 0855 Oslo, Norway; hakon.valen@niom.no

[3] School of Dentistry, Institute of Clinical Sciences, College of Medical and Dental Sciences, University of Birmingham, Birmingham B15 2TT, UK; j.camilleri@bham.ac.uk

* Correspondence: andreas.koutroulis@odont.uio.no (A.K.); p.t.sunde@odont.uio.no (P.T.S.)

a Dentin segments treated with 17% EDTA – No material application

1

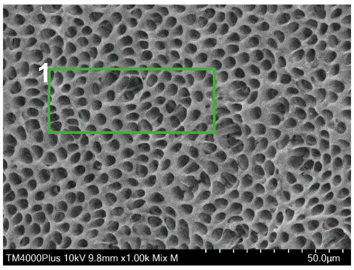

Element	At No.	Netto	Mass [%]	Mass Norm. [%]	Atom [%]	abs. error [%] (1 sigma)	rel. error [%] (1 sigma)
C	6	31173	34.50	42.10	56.78	4.26	12.34
N	7	1911	8.99	10.98	12.69	1.75	19.43
O	8	6843	13.82	16.87	17.08	2.08	15.02
Na	11	1558	0.84	1.02	0.72	0.09	10.39
P	15	39419	6.91	8.43	4.41	0.29	4.21
Ca	20	64207	16.87	20.60	8.32	0.53	3.15
			Sum 81.93	100.00	100.00		

2

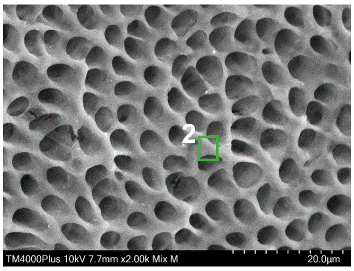

Element	At No.	Netto	Mass [%]	Mass Norm. [%]	Atom [%]	abs. error [%] (1 sigma)	rel. error [%] (1 sigma)
C	6	2214	6.72	7.58	13.64	1.18	18.90
O	8	11293	30.52	34.44	49.68	4.23	13.85
Mg	12	742	0.27	0.31	0.29	0.05	16.59
Al	13	1705	0.51	0.57	0.49	0.05	10.68
Si	14	1082	0.28	0.32	0.26	0.04	14.57
P	15	54450	14.21	16.04	11.95	0.57	4.01
Ca	20	80746	35.76	40.36	23.24	1.10	3.07
			Sum 88.12	100.00	100.00		

b

TZ-base

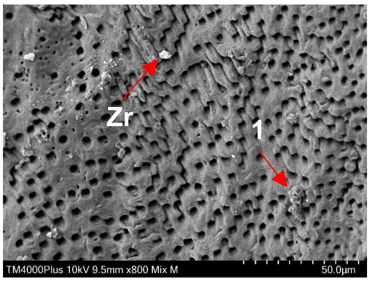

1

Element	At No.	Netto	Mass [%]	Mass Norm. [%]	Atom [%]	abs. error [%] (1 sigma)	rel. error [%] (1 sigma)
C	6	10184	20.37	17.35	27.04	2.87	14.09
N	7	1710	8.07	6.87	9.18	1.61	19.98
O	8	23229	47.07	40.09	46.90	5.97	12.68
Mg	12	3068	0.71	0.61	0.47	0.07	9.48
Si	14	4104	0.76	0.65	0.43	0.06	7.97
P	15	39382	7.77	6.62	4.00	0.32	4.17
Ca	20	64591	28.14	23.97	11.19	0.87	3.09
Zr	40	13815	4.52	3.85	0.79	0.20	4.44
			Sum 117.42	100.00	100.00		

2

TZ-bg20

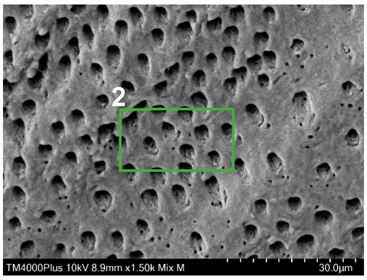

Element	At No.	Netto	Mass [%]	Mass Norm. [%]	Atom [%]	abs. error [%] (1 sigma)	rel. error [%] (1 sigma)
C	6	11534	22.33	18.27	28.25	3.09	13.84
N	7	1607	8.09	6.62	8.78	1.64	20.29
O	8	22757	48.92	40.03	46.48	6.21	12.70
Na	11	881	0.30	0.24	0.20	0.05	16.45
Mg	12	2049	0.50	0.41	0.31	0.06	11.27
P	15	37190	8.30	6.79	4.07	0.34	4.15
Ca	20	69916	29.54	24.17	11.20	0.91	3.09
Zr	40	11538	4.24	3.47	0.71	0.19	4.51
			Sum 122.22	100.00	100.00		

TZ-bg40

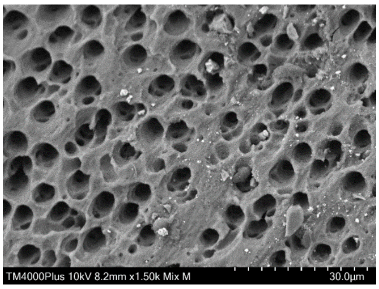

c

TZ-Ag1

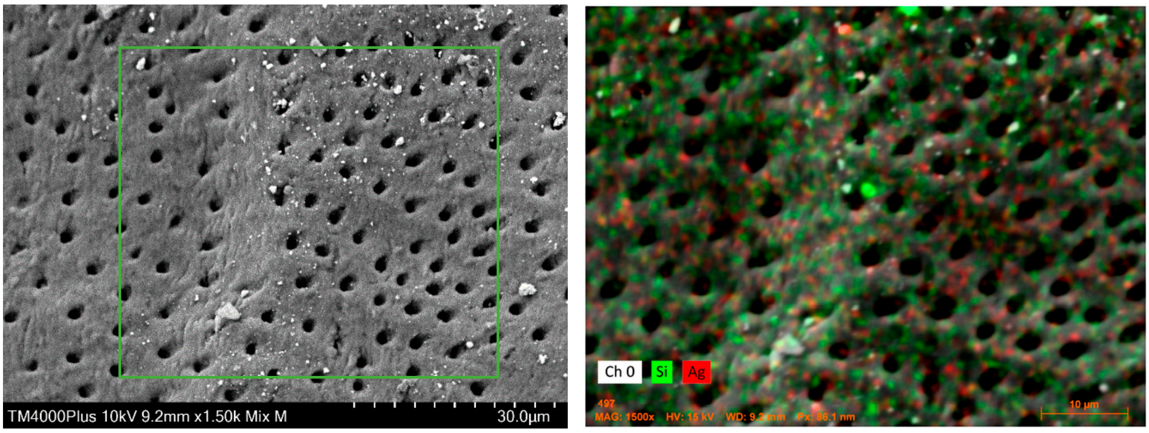

TZ-Ag2

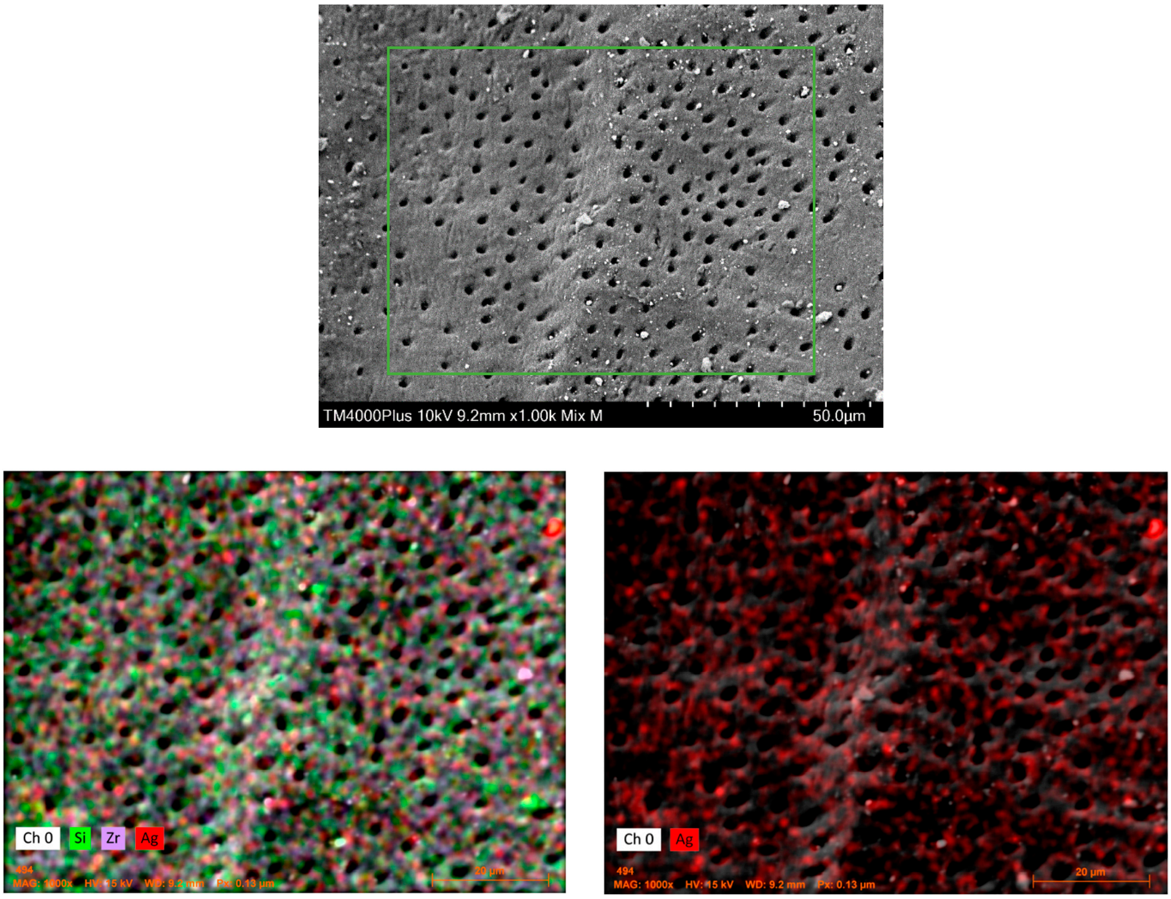

d

Biodentine **1**

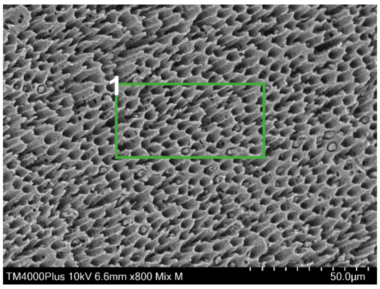

Element	At No.	Netto	Mass [%]	Mass Norm. [%]	Atom [%]	abs. error [%] (1 sigma)	rel. error [%] (1 sigma)
C	6	3112	7.65	8.37	14.93	1.34	17.50
O	8	17139	38.34	41.97	56.20	5.03	13.12
Mg	12	1306	0.37	0.40	0.35	0.05	13.48
Al	13	4762	1.12	1.22	0.97	0.08	7.33
Si	14	2307	0.50	0.54	0.41	0.05	10.07
P	15	44148	9.76	10.69	7.39	0.40	4.10
Cl	17	2530	0.73	0.80	0.48	0.06	7.56
Ca	20	77337	32.90	36.01	19.25	1.01	3.07
			Sum 91.36	100.00	100.00		

TotalFill **2**

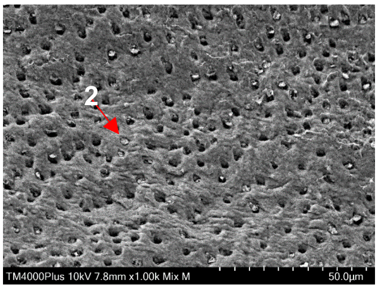

Element	At No.	Netto	Mass [%]	Mass Norm. [%]	Atom [%]	abs. error [%] (1 sigma)	rel. error [%] (1 sigma)
C	6	2414	7.36	7.62	15.03	1.36	18.48
O	8	17087	36.62	37.93	56.18	4.81	13.13
Mg	12	4119	1.42	1.47	1.43	0.11	7.57
Si	14	19289	5.04	5.23	4.41	0.24	4.77
P	15	16885	4.55	4.71	3.61	0.20	4.48
Ca	20	49816	23.78	24.63	14.56	0.74	3.12
Zr	40	40062	17.77	18.41	4.78	0.70	3.92
			Sum 91.36	100.00	100.00		

IRM

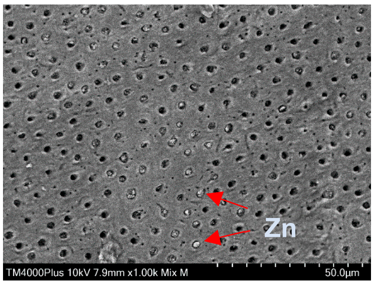

Figure S1. Indicative scanning electron microscope (SEM) images of dentin surfaces from the split tooth block model accompanied by energy dispersive x-ray analyses (EDX) of selected regions or elemental mapping. Images suggest that no complete coverage of the dentin surface by material remnants takes place following separation of the two parts of the root segment. (**a**) Root surfaces treated with 17% ethylenediaminetetraacetic acid (EDTA) and no application of material (control specimens) were assessed to detect the main elements as well as trace ones depicted in dentin segments following EDTA pretreatment. (**b**) SEM images and EDX analyses from dentin surfaces previously in contact with TZ-base, TZ-bg20 and TZ-bg40. Some zirconium oxide particles as well as cement particles can be sporadically depicted. (**c**) Dentin surfaces and elemental maps for TZ-Ag1 and TZ-Ag2. Some aggregated silver nanoparticles could also be depicted (red arrow). (**d**) SEM images and EDX analyses from dentin surfaces previously in contact with Biodentine, TotalFill and IRM. Material residues are mainly depicted inside the dentin tubules.

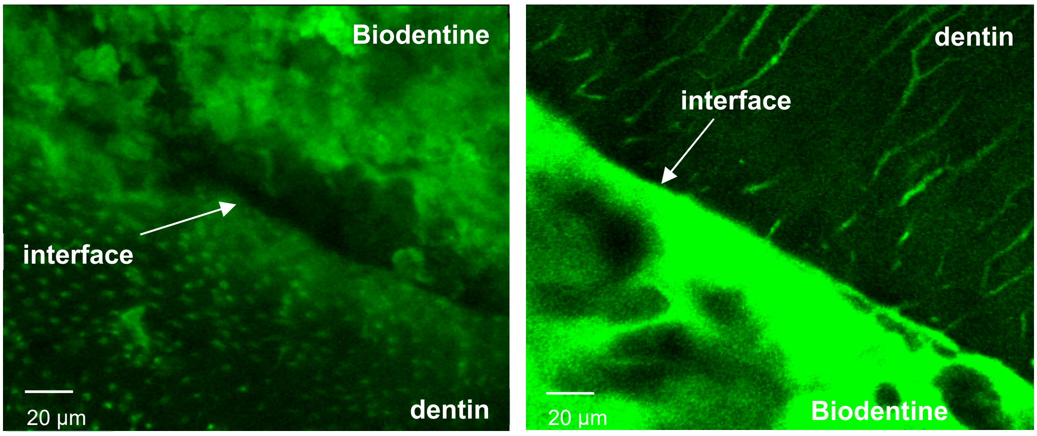

Figure S2. Representative confocal laser scanning microscope images (60×) for orientation upon the dentin/material surface and optimization of threshold options. Specimens were not exposed to any bacteria. Biodentine was used for these internal controls.
